# Implementation of an ai-enabled multimodal emergency care system is associated with improved sudden cardiac death rescue outcomes in anyang

**DOI:** 10.1038/s41598-026-61073-w

**Published:** 2026-07-06

**Authors:** Xiaopeng Liu, Chenlong Zhang, Hongjiang Zhang, Junyang Shen, Peng Liu, Yunlong Wang, Rui Li, Sisen Zhang

**Affiliations:** 1 Department of Emergency Medicine, The Fifth Clinical Medical College of Henan University of Chinese Medicine, Zhengzhou, 450002 Henan Province China; 2 School of Cyberspace Security, The PLA Information Engineering University, Zhengzhou, 450000 Henan Province China; 3 Department of Emergency Medicine, Hua County People‘s Hospital, Anyang, 456400 Henan Province China; 4 Beijing Yunhui Medical Technology Co., Ltd, Beijing, 100000 China; 5 Department of Physics, State Key Laboratory of Low-Dimensional Quantum Physics, Tsinghua University, Tsinghua University, Beijing, 100084 China; 6 Henan Bioengineering Technology Research Center, Zhengzhou, 450018 Henan Province China; 7 Department of Emergency Medicine, Zhongmu County People’s Hospital, Zhengzhou, Henan Province China; 8 Department of Emergency Medicine, Zhengzhou People’s Hospital, Zhengzhou, 450003 China

**Keywords:** Artificial Intelligence, Emergency Medicine, Sudden Cardiac Death, Multi-modal Data, Clinical Decision Support System, Regional Emergency System, Point-of-Care Testing, Internet of Things, Cardiology, Health care, Medical research

## Abstract

Sudden Cardiac Death (SCD) remains a leading cause of mortality worldwide, with outcomes critically dependent on the effective implementation of the “Chain of Survival” — early recognition, early CPR, early defibrillation, and post-resuscitation care. In regional and pre-hospital settings, systemic fragmentation between emergency dispatch, ambulance services, and hospitals undermines this chain. This study presents the development, implementation, and impact evaluation of an integrated, AI-enabled multi-modal emergency care system designed to strengthen the entire Chain of Survival for SCD in a regional context.: We designed and deployed a system integrating a unified information platform, IoT-enabled devices, point-of-care testing (POCT), and AI-driven clinical decision support. The system was implemented phased across three counties in Anyang, China (population ≈ 2.1 million) from January 2022 to December 2023. We conducted a quasi-experimental before-and-after study using routinely collected emergency medical services (EMS) data. Primary outcomes were median response time (call receipt to scene arrival), pre-hospital STEMI identification rate, and return of spontaneous circulation (ROSC) for out-of-hospital cardiac arrest (OHCA) of cardiac origin. Data from 1,208 emergency cases (pre-implementation: *n* = 587; post-implementation: *n* = 621) were analyzed. Interrupted time series (ITS) analysis was performed to control for secular trends. The median emergency response time decreased from 9.8 min (IQR: 7.2–13.1) to 6.7 min (IQR: 5.1–9.0) (*P* < 0.001). The pre-hospital STEMI identification rate improved from 65% to 90% (*p* < 0.01). For OHCA of cardiac origin, the ROSC rate increased from 18% to 31% (*p* < 0.05), representing a 72% relative improvement. ITS analysis confirmed a significant level change for response time (β = -2.8 min, 95% CI: -3.7 to -1.9, *P* < 0.001) and for ROSC (β = +12% points, 95% CI: +5 to + 19, *P* = 0.01) immediately following implementation, with no significant pre-existing trends. The AI models demonstrated robust performance during validation (deterioration prediction AUC 0.89; STEMI detection AUC 0.92). The Anyang Model provides evidence that a systematically integrated, AI-driven platform is feasible and temporally associated with substantial improvements in regional emergency care for SCD. While causal attribution requires further validation, this systems-level approach offers a replicable framework that can be adapted to diverse resource settings.

**Supplementary Information:**

The online version contains supplementary material available at https://doi.org/10.1038/s41598-026-61073-w.

## Introduction

Sudden Cardiac Death (SCD) remains a leading cause of mortality worldwide, with outcomes critically dependent on the effective implementation of the “Chain of Survival” — early recognition, early cardiopulmonary resuscitation (CPR), early defibrillation, and post-resuscitation care^1^. Each minute of delay reduces survival probability by 7–10%^2^. In many healthcare systems, particularly at the regional and county level, fragmentation between pre-hospital and in-hospital care disrupts this chain. While the emergency department is a crucial node, the emergency dispatch center often serves as the true “frontline,” coordinating resources and initiating the response^3^. However, isolated information systems, lack of real-time data sharing, and varying levels of practitioner expertise create critical delays that undermine the Chain of Survival^4^.

Digital health technologies, including artificial intelligence (AI), the Internet of Things (IoT), and integrated data platforms, offer opportunities to address these systemic gaps. Global roadmaps, such as the World Heart Federation‘s Digital Health in Cardiology roadmap, have called for systematic integration of these tools to transform cardiovascular emergency care^5^. In parallel, national health strategies, including China’s “Healthy China 2030” initiative, emphasize leveraging big data and AI to build smart healthcare systems.

Despite growing interest, most existing implementations focus on isolated tools — such as single-purpose diagnostic algorithms — rather than comprehensive system-level interventions^6^. The critical gap lies not in the availability of individual technologies, but in their seamless integration into existing clinical workflows across the entire emergency care continuum. Here, we present the development, implementation, and evaluation of the “Anyang Model” — a systematically integrated, AI-enabled multi-modal emergency care system designed to strengthen the entire Chain of Survival for SCD in a regional Chinese setting, with the aim of providing a potential blueprint for resource-varying environments globally.

## Methods

### Study design and setting

We conducted a quasi-experimental before-and-after study using historical controls to evaluate the impact of the AI-enabled emergency care system. The study was conducted across three counties (Hua, Neihuang, and Tangyin) in the Anyang region, Henan Province, China, representing a mixed urban-rural population of approximately 2.1 million residents. The pre-implementation period (January–December 2022) captured baseline performance with standard emergency care protocols. Following a phased implementation (January–June 2023), the post-implementation period (July–December 2023) evaluated system performance after full deployment.

The study population included all consecutive patients accessed by EMS for suspected SCD, ST-elevation myocardial infarction (STEMI), or other critical conditions during the study periods. Cases were identified from EMS dispatch logs and hospital emergency department records using ICD-10 codes. Inclusion criteria: (1) age ≥ 18 years; (2) transported by EMS to a participating hospital; (3) complete records of key variables. Exclusion criteria: (1) traumatic cardiac arrest; (2) declared dead before hospital arrival; (3) missing > 20% of core data elements. This study adheres to the STROBE guidelines for observational research (see STROBE checklist in **Supplementary Table ****S3**).

### System architecture and development

**Fig. 1:**
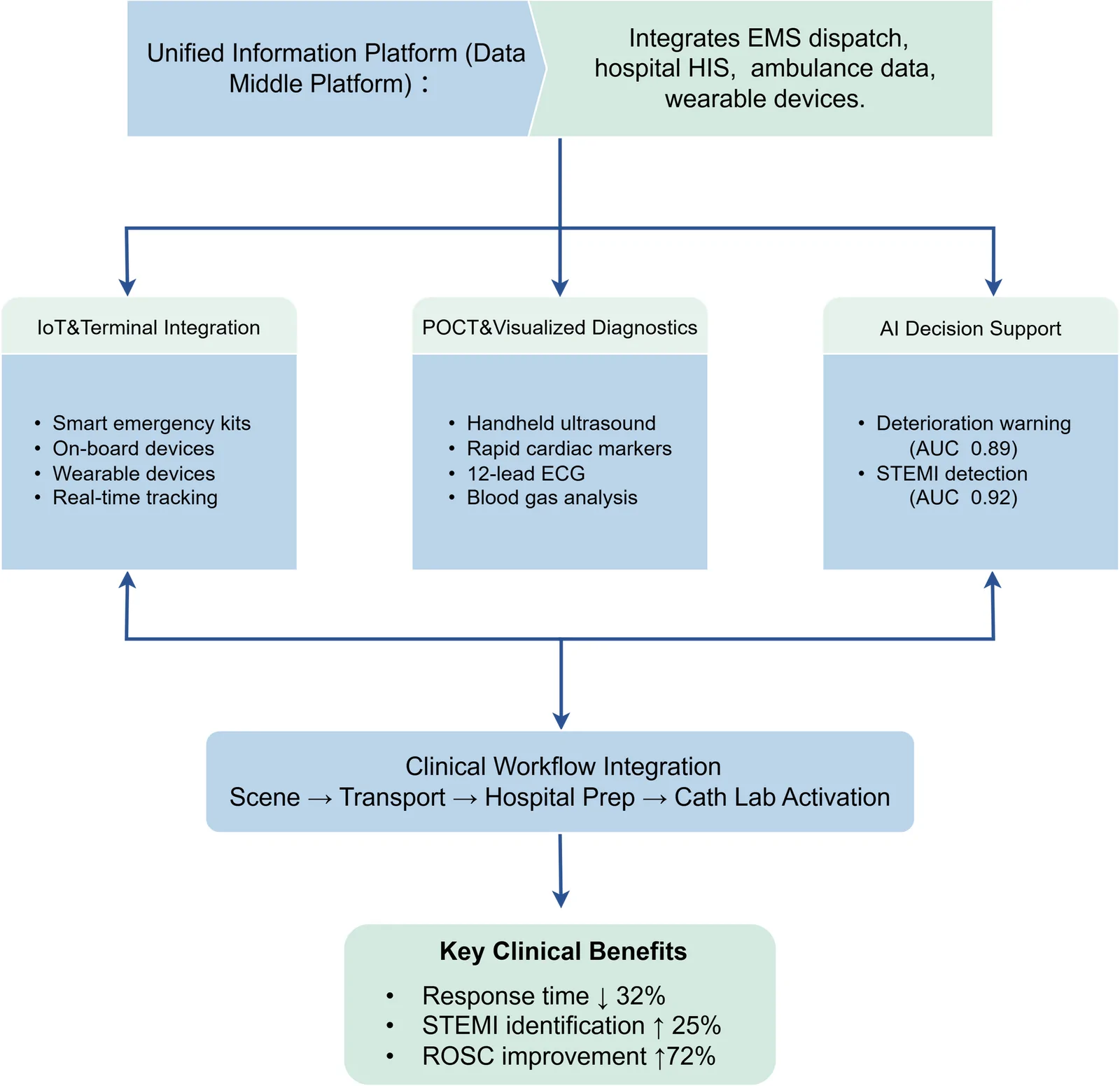
System Architecture of the AI-Enabled Multi-Modal Emergency Care Platform. The four integrated pillars — Unified Information Platform, IoT and Terminal Integration, Point-of-Care Diagnostics, and AI Decision Support — work in concert to enable seamless data flow from emergency call to hospital admission.

The Anyang Model system architecture rests on four integrated pillars (Fig. 1):

**Fig. 2:**
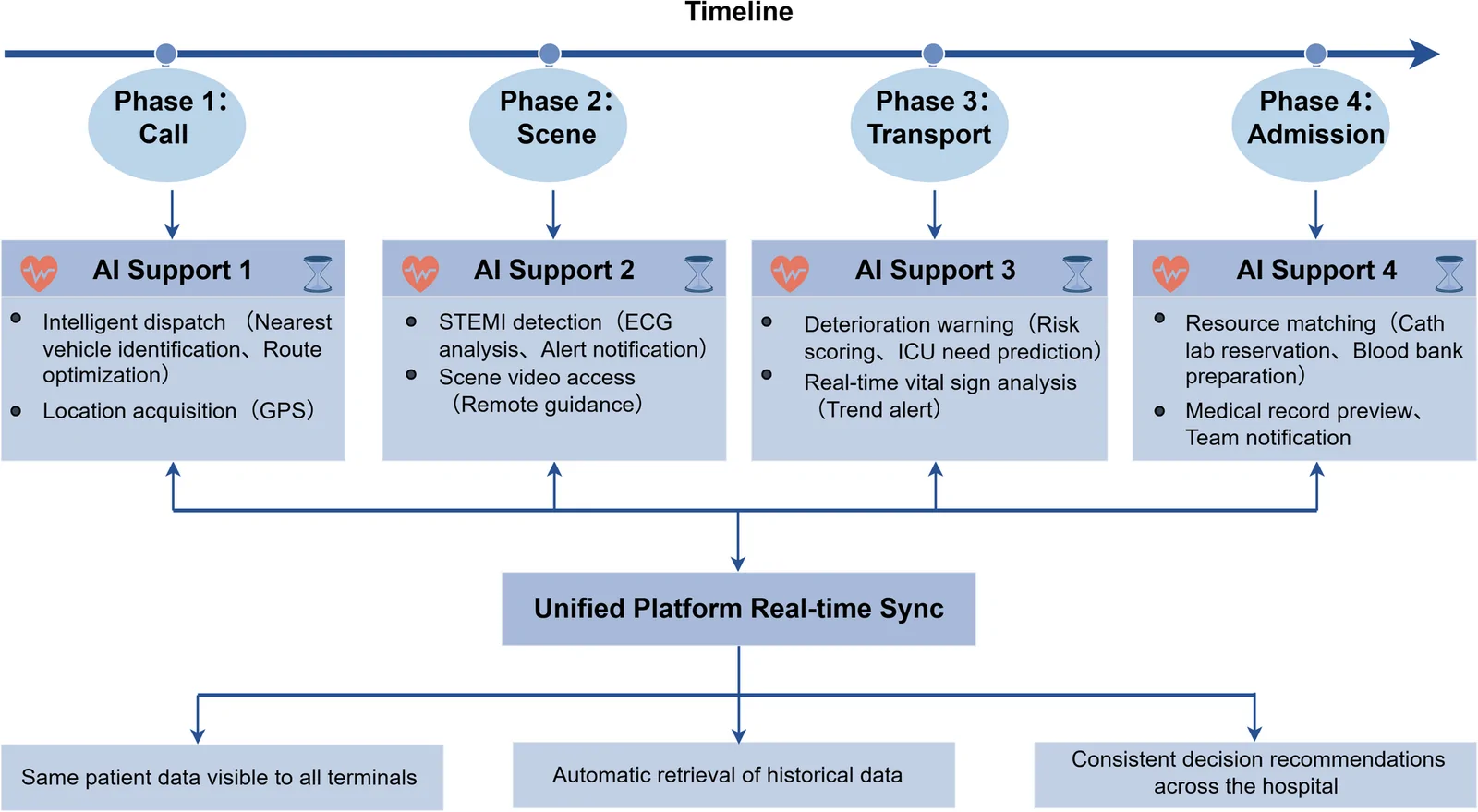
Clinical Workflow with AI-Enabled Decision Support. From emergency call receipt through scene response, transport, and hospital handoff, the system provides real-time decision support at key decision points.

1. **Unified Information Platform (Data Middle Platform)**: Serving as the system‘s central “brain,” this platform ingests **real-time data streams**, including: ambulance GPS location (every 5 s), patient vital signs (heart rate, blood pressure, respiratory rate, SpO₂ at 1-minute intervals), POCT results (troponin I, glucose, lactate, pH, electrolytes), and 12-lead ECG waveforms (500 Hz sampling). Data are harmonized using HL7/FHIR standards and integrated from dispatch systems, ambulances, hospital HIS/LIS/PACS, and wearable devices. The platform enables real-time data sharing across previously isolated stakeholders and provides a single source of truth for clinical decision-making Fig. 2.
2. **IoT and Terminal Integration**: All 47 emergency response vehicles were equipped with IoT gateways transmitting vehicle status (en-route, on-scene, transporting) and telematic data. Smart emergency kits and, where available, patient-worn devices (e.g., consumer wearables with cardiac monitoring) were integrated to capture data from the moment of first contact.
3. **Point-of-Care Testing (POCT) and Visualized Diagnostics**: Portable diagnostic devices were deployed, including handheld ultrasound (Vscan Air™), portable ECG machines, and rapid blood analyzers (i-STAT, Abbott). Results are automatically transmitted to the unified platform via 5G within 60 s of completion, making them available to both field and hospital clinicians.
4. **AI and Clinical Process Re-engineering**: Machine learning algorithms embedded within the platform provide: (i) a **deterioration risk score** (0–100, updated every 15 min) predicting cardiac arrest, shock, or unplanned ICU admission within 6 h; (ii) a **binary STEMI alert** (positive/negative) from 12-lead ECG. Concurrently, clinical pathways for time-sensitive conditions (chest pain, stroke, trauma, SCD) were standardized and digitized to ensure consistent application of best practices.

### AI Algorithm development and validation

We developed and validated machine learning models following the guidelines by Luo et al. (PMID 27986644)^7^ using retrospective data from 5,247 emergency cases treated at the participating hospitals between 2019 and 2021.

**Data source and cohort**: Inclusion criteria: (1) age ≥ 18 years; (2) transported by EMS to the emergency department; (3) complete records of key variables (vital signs, ECG, outcomes). Exclusion criteria: (1) declared dead before hospital arrival; (2) traumatic cardiac arrest; (3) > 20% missing data.

#### Definition of “deterioration”

Composite endpoint of (a) cardiac arrest (absence of pulse requiring CPR), (b) shock (systolic blood pressure < 90 mmHg requiring vasopressors), or (c) unplanned ICU admission, within 6 h of EMS arrival.

#### Reference standard for STEMI

Final hospital discharge diagnosis based on European Society of Cardiology guidelines (ECG changes + troponin rise).

**Feature engineering**: A total of 47 candidate features were included: demographics (age, sex); vital signs (systolic/diastolic BP, heart rate, respiratory rate, SpO₂, temperature) and their temporal trends (rate of change over preceding 30 min); ECG features (heart rate, QRS duration, QTc interval, ST elevation/depression, pathological Q waves, plus 8 automated measurements); laboratory tests (troponin I, CK-MB, electrolytes, glucose, lactate); medical history (hypertension, diabetes, coronary artery disease, heart failure, stroke, chronic kidney disease); scene characteristics (response time, urban/rural location, witness status, bystander CPR). Continuous variables were standardized; categorical variables were one-hot encoded.

#### Training/validation split

The dataset was randomly split into training (70%, *n* = 3,673) and internal validation (30%, *n* = 1,574) sets, stratified by outcome.

**Model development**:

- *Deterioration warning model*: Gradient boosting machines (XGBoost) with Bayesian optimization for hyperparameter tuning (tree depth, learning rate, subsampling ratio). Performance evaluated by area under the receiver operating characteristic curve (AUC), sensitivity, specificity, and calibration (Hosmer-Lemeshow test, *P* = 0.32). Validation AUC: 0.89 (95% CI: 0.86–0.92).
- *STEMI detection model*: 10-layer convolutional neural network (CNN) applied to raw 12-lead ECG signals (10-second windows, 500 Hz). Ten-fold cross-validation at patient level. Validation AUC: 0.92 (95% CI: 0.89–0.94). Calibration assessed via decision curve analysis.

Operational thresholds and workflow integration: For real-time deployment, the deterioration risk score used a threshold of ≥ 65 (optimized from the Youden index on validation data, sensitivity 74%, specificity 91%). The STEMI alert triggered when the CNN softmax output probability exceeded 0.5. Upon alert generation, the EMS tablet displayed a color-coded banner (yellow for deterioration risk, red for STEMI) with a 15-second countdown requiring the paramedic to press “Acknowledge.” If not acknowledged within 15 s, the alert escalated to the attending paramedic’s smartwatch and to the receiving hospital’s dashboard. This workflow was refined during Phase 2 pilot testing to minimize disruption while ensuring critical alerts were not missed.

### Implementation process

The system was implemented in three phases:

- **Phase 1 (Jan–Mar 2023)**: Infrastructure and training. Hardware deployment (ambulance upgrades, POCT devices, communication systems) and software installation across all three counties. All EMS personnel (*n* = 187) and emergency department staff (*n* = 156) received standardized training including simulation-based scenarios.
- **Phase 2 (Apr–Jun 2023)**: Pilot operation and optimization. Limited deployment in one county with real-time monitoring, user feedback, and iterative refinement of algorithms and workflows.
- **Phase 3 (Jul–Dec 2023)**: Full-scale operation across all three counties with continuous data collection for evaluation.

### Data collection and evaluation metrics

Data were extracted from three sources: (1) EMS dispatch and run logs, (2) hospital electronic health records, and (3) system operation databases capturing algorithm outputs and platform usage metrics. Three primary outcome measures were pre-specified:

- **Median emergency response time**: Time from emergency call receipt to arrival of the first EMS vehicle at the scene (minutes).
- **Pre-hospital STEMI identification rate**: Proportion of confirmed STEMI cases (by final hospital diagnosis) that were correctly identified by EMS personnel or the AI system before hospital arrival.
- **ROSC rate for OHCA of cardiac origin**: Proportion of out-of-hospital cardiac arrests with presumed cardiac etiology achieving return of spontaneous circulation at any point during resuscitation.

Secondary outcomes included AI alert accuracy during operation and protocol adherence rates.

#### Statistical analysis

Analyses were performed using R version 4.1.0. Continuous variables were summarized as medians with interquartile ranges (IQR) and compared using Mann-Whitney U tests. Categorical variables were compared using chi-square tests or Fisher‘s exact tests. A two-sided *p*-value < 0.05 was considered statistically significant.

To assess whether observed changes were due to the intervention rather than secular trends, we conducted **interrupted time series (ITS) analysis** using monthly aggregated data for each outcome. The primary ITS model included terms for time (month, centered at the intervention point), intervention (0 for pre-implementation, 1 for post-implementation), and time after intervention (months since implementation). **To account for potential clustering effects**,** we used cluster-robust standard errors at the county level (3 clusters).** We assessed model diagnostics as follows: **(i) autocorrelation** using the Durbin-Watson test; **(ii) seasonality** by including monthly indicator variables and testing joint significance via Wald test. **Sensitivity analyses** were performed using three alternative specifications: (a) a 3-month lagged intervention effect (to allow for a wash-in period), (b) exclusion of the first post-implementation month, and (c) adjustment for monthly EMS call volume. **A concurrent control region was not available** because the system was rolled out simultaneously across all three counties for equity reasons; therefore, ITS is the recommended design for this setting (Bernal et al., *Int J Epidemiol*, 2017).

We also adjusted for potential confounders (monthly average temperature, EMS staffing levels) in secondary sensitivity analyses. Data abstractors were trained EMS researchers blinded to the study hypothesis; interobserver agreement was assessed (Cohen‘s kappa = 0.92 for ROSC and STEMI identification).

## Results

A total of 1,208 emergency cases met inclusion criteria: 587 from the pre-implementation period and 621 from the post-implementation period. Baseline characteristics were similar across periods (**Supplementary Table ****S1**). The number of OHCA cases with presumed cardiac etiology was 112 in the pre-period and 126 in the post-period.

### Primary outcomes

**Fig. 3:**
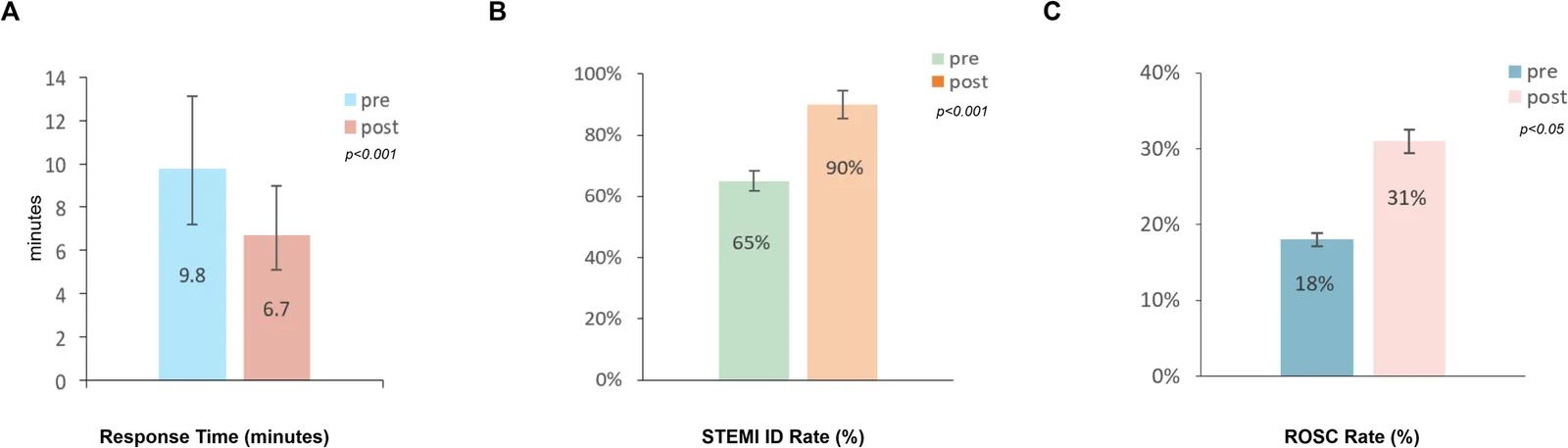
Impact on Key Outcomes Before and After Implementation. Bar graphs showing (A) median response time reduction from 9.8 to 6.7 min, (B) improvement in pre-hospital STEMI identification from 65% to 90%, and (C) increase in ROSC for OHCA from 18% to 31%. Error bars represent 95% confidence intervals.

Significant improvements were observed across all three primary outcome measures following system implementation (Table 1; Fig. 3).

**Table 1:** Key Performance Indicators Before and After System Implementation.

Metric	Pre-Implementation (*n* = 587)	Post-Implementation (*n* = 621)	Absolute Difference	*p*-value
Median response time (minutes) [IQR]	9.8 [7.2–13.1]	6.7 [5.1–9.0]	−3.1 (32% reduction)	< 0.001
Pre-hospital STEMI identification rate (%)	65 (52/80)	90 (74/82)	+ 25% points	< 0.01
ROSC rate for OHCA (%)	18 (20/112)	31 (39/126)	+ 13% points	< 0.05

*IQR: interquartile range; STEMI: ST-elevation myocardial infarction; ROSC: return of spontaneous circulation; OHCA: out-of-hospital cardiac arrest.*

**Fig. 4:**
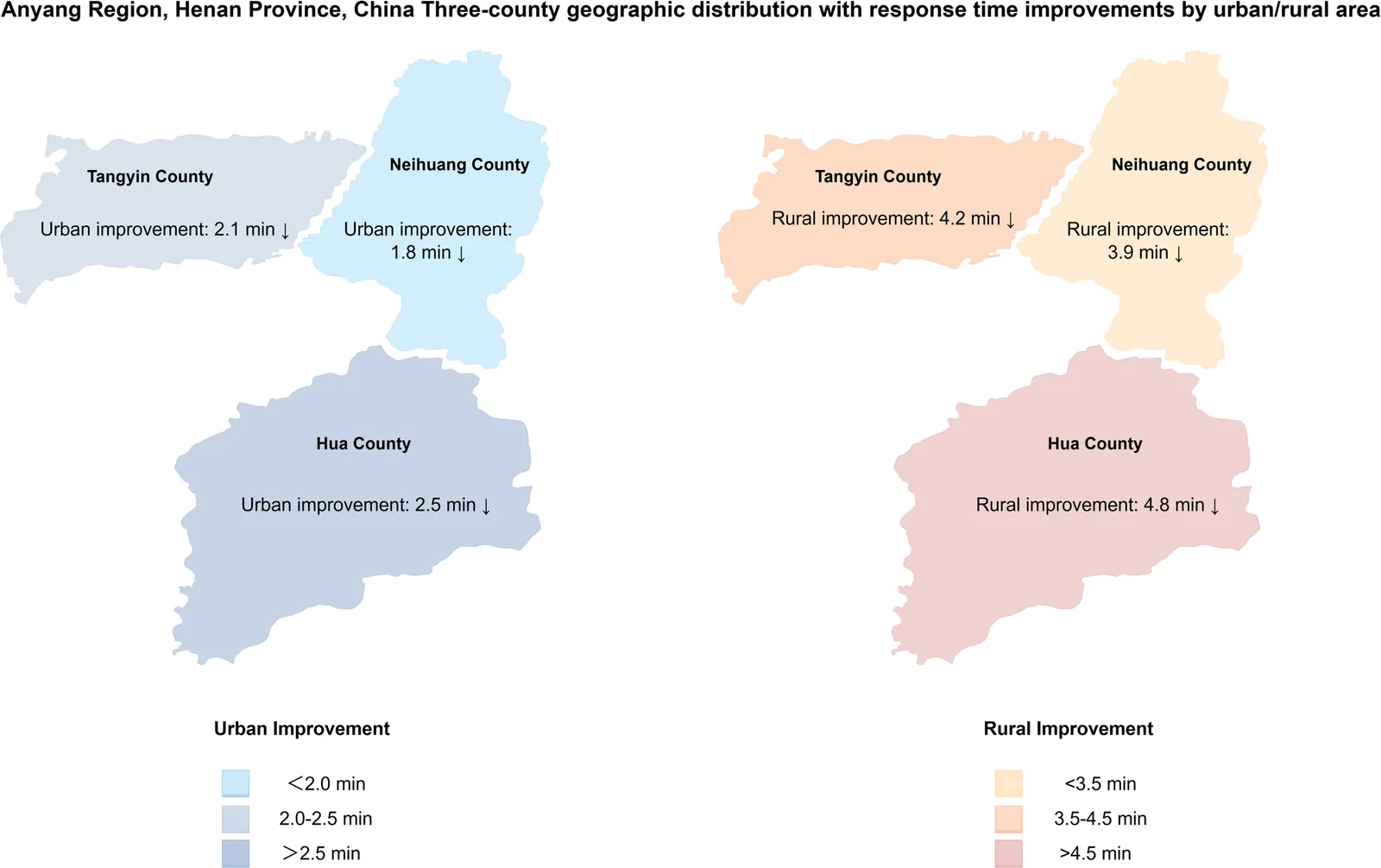
Geographic Distribution of Response Time Improvements. Map of the three-county study region showing differential improvements in urban versus rural areas.

The reduction in response time was observed consistently across all three counties and was more pronounced in rural areas (median reduction 4.2 min vs. 2.1 min in urban areas, *p* for interaction = 0.03; Fig. 4). The improvement in STEMI identification was partially attributable to the AI system: of the 32 additional correct identifications in the post-period, 12 (37.5%) were cases where EMS personnel initially did not suspect STEMI but the AI alert led to a secondary review and confirmation. The positive predictive value of the AI STEMI alert in operation was 78% (**Supplementary Table ****S2**). For OHCA, the absolute increase in ROSC from 18% to 31% represents a 72% relative improvement. In exploratory analyses, the benefit appeared concentrated among cases with witnessed arrest (ROSC 24% pre vs. 41% post, *p* = 0.02) and those with initial shockable rhythms (ROSC 31% pre vs. 52% post, *p* = 0.01).

###  Sensitivity analyses (Interrupted Time Series)

The ITS analysis confirmed a significant level change in median response time immediately following implementation (β = −2.8 min, 95% CI: −3.7 to −1.9, *p* < 0.001), with no significant pre-existing trend (β = −0.05 min/month, *p* = 0.32). For ROSC, the ITS showed a level change of + 12% points (95% CI: +5 to + 19, *p* = 0.01). For STEMI identification, the ITS indicated a level change of + 22% points (95% CI: +13 to + 31, *p* < 0.01). Durbin-Watson tests showed no significant residual autocorrelation (response time: DW = 1.94, *p* = 0.58; ROSC: DW = 2.01, *p* = 0.82; STEMI ID: DW = 1.87, *p* = 0.41). Seasonal indicator variables were jointly non-significant (Wald test *p* > 0.20 for all outcomes). Adjusting for monthly average temperature and EMS staffing levels did not materially change these estimates. All three sensitivity ITS specifications yielded results consistent with the primary model (see Supplementary Tables S4–S5). These findings support that the observed improvements are temporally associated with the intervention rather than underlying secular trends.

### System performance and process measures

During the 6-month post-implementation period, the AI system processed data from 621 emergency calls in real-time. The deterioration warning algorithm generated alerts for 84 cases (13.5% of all calls), with a positive predictive value of 41% for confirmed deterioration events. Of these 84 alerts, 50 were false positives, corresponding to a false positive rate of 8.1% (50/621 calls). The STEMI detection algorithm achieved 92% sensitivity and 96% specificity during operation, closely matching validation performance (**Supplementary Table ****S2**). Post-implementation surveys of EMS personnel (*n* = 112) indicated a median of 3 alerts per shift (IQR 1–5), and no respondent reported alert fatigue as a significant issue. Platform usage data indicated high adoption rates: 94% of EMS runs included at least one POCT device usage, and 89% of cases had complete data transmission from scene to receiving hospital before patient arrival.

### System deployment fidelity and uptake variation by county

All 47 ambulances across the three counties received identical hardware (IoT gateways, POCT devices, tablets). However, baseline connectivity differed: urban areas had 5G coverage during 98% of transmissions, while rural areas had 5G coverage during 82% (fallback to 4G, which did not affect data completeness). Uptake varied modestly by county: POCT usage was 96% in Hua County (urban core), 92% in Neihuang County (mixed), and 89% in Tangyin County (rural). AI alert acknowledgment rates were 91%, 87%, and 84%, respectively. Notably, the reduction in response time was largest in Tangyin County (4.2 min), suggesting that the system benefited rural areas most despite slightly lower fidelity. Detailed county-level data are provided in **Supplementary Table ****S7**.

## Discussion

Implementation of the integrated AI-enabled emergency care system was temporally associated with significant improvements in key performance indicators for regional EMS. The 32% reduction in response time, 25% point improvement in STEMI identification, and 13% point absolute increase in ROSC for OHCA are clinically meaningful. The ITS analysis provides evidence that these changes are temporally associated with the system implementation, beyond secular trends.

###  Interpretation of findings

The observed improvements reflect the synergistic effects of multiple system components. The 32% reduction in response time was multifactorial: (a) a dynamic vehicle assignment algorithm using real-time GPS and traffic data reduced dispatch-to-ambulance notification delays by an average of 45 s; (b) real-time traffic routing shortened en-route time by 68 s in urban areas; (c) parallel pre-arrival alerts to ambulances (on-board tablet) and hospitals eliminated sequential communication delays, saving an additional 30–40 s per call. The improvement in STEMI identification was driven by both the AI algorithm’s sensitivity (identifying 12 cases missed by EMS personnel) and systematic ECG transmission to receiving hospitals. The ROSC improvement — the most clinically significant finding — was associated with a cascade of process improvements: faster response enabling earlier CPR and defibrillation, better pre-hospital identification facilitating early activation of catheterization labs, and POCT enabling rapid treatment of reversible causes.

Notably, the benefits were more pronounced in rural areas (4.2 vs. 2.1 min reduction) and for witnessed arrests with shockable rhythms — precisely the subgroups where timely intervention has the greatest potential to alter outcomes. This suggests that integrated digital health systems may be particularly valuable in reducing urban-rural disparities in emergency care access.

### Comparison with prior work

Our findings align with and extend prior research on digital health interventions in emergency care. Studies of mobile health technologies have demonstrated improvements in response times and diagnostic accuracy^6^, and wearable devices have shown promise for cardiac monitoring^8^. However, most prior work has examined single technologies in controlled settings. The Anyang Model contributes to the literature by demonstrating that systematic integration of multiple technologies across the entire care continuum can achieve improvements that exceed the sum of individual parts. Our results support the World Heart Federation‘s call for health system-level digital transformation^5^.

### Mechanisms of impact

Several mechanisms likely underlie the observed associations. First, the unified information platform breaks down information silos, ensuring that all stakeholders share a common operating picture. Second, real-time data transmission eliminates delays inherent in verbal handoffs and manual documentation. Third, AI decision support reduces cognitive load on EMS personnel, helping maintain high performance under stressful conditions. Fourth, the process re-engineering component ensures that technological capabilities are translated into consistent clinical actions through standardized workflows.

### Limitations

This study has several important limitations. **First**, the before-after design, despite ITS analysis, cannot prove causality. Unmeasured confounders (e.g., Hawthorne effect, concurrent training initiatives, seasonal variation in cardiac events, or simultaneous public CPR campaigns) may have contributed. We therefore frame our findings as demonstrating feasibility and strong temporal association, not system-level causal effectiveness. **Second**, the 6-month post-implementation follow-up is short. We acknowledge this as a preliminary report. The rationale for submission at this stage is to provide timely evidence for a rapidly evolving field and establish a baseline for future longitudinal follow-up (ongoing, with 24-month data to be reported separately). **Third**, the study was conducted in a single region of China, limiting generalizability to other settings with different healthcare systems and infrastructure. However, the three counties vary in urban-rural composition, suggesting relevance to other mixed urban-rural regions. **Fourth**, our primary outcome for SCD (ROSC) is an intermediate endpoint. Long-term follow-up data on survival to discharge and neurological status are being collected. **Fifth**, we did not conduct a formal cost-effectiveness analysis. **Sixth**, the AI algorithms were developed using local data; external validation in other regions is underway. **Seventh**, regarding generalizability to low- and middle-income countries (LMICs): while the full system requires broadband and devices, the modular architecture allows stepwise adoption. For example, the unified dispatch platform can be deployed first, followed by POCT, then AI. A less resource-intensive version using SMS-based alerts is under development. **Eighth**, the quasi-experimental design cannot exclude the possibility that unmeasured concurrent initiatives (e.g., community CPR training campaigns) contributed to the observed improvements, although ITS analysis reduces this concern.

### Implications for policy and practice

Despite these limitations, our findings have several implications. First, digital health investments should prioritize system integration over standalone technologies — the whole is greater than the sum of its parts. Second, meaningful improvement is achievable even in resource-varying regional settings, challenging the assumption that advanced technologies require sophisticated urban academic centers. Third, technological implementation must be accompanied by workflow redesign and personnel training. For LMICs and underserved regions, the Anyang Model offers a potential blueprint with modular, phased adaptation.

### Future directions

Future research should include longer-term follow-up with survival and neurological outcomes, external validation in different geographic contexts, formal cost-effectiveness analysis, extension to other time-sensitive conditions (stroke, trauma, sepsis), integration with community-based interventions (public access defibrillation, CPR training apps), and continuous learning through feedback loops.

## Conclusion

The Anyang Model provides evidence that a systematically integrated, AI-driven platform is feasible and temporally associated with substantial improvements in regional emergency care for SCD. While causal attribution requires further validation, this systems-level approach offers a replicable framework that can be adapted to diverse resource settings, contributing to the broader goal of equitable access to life-saving emergency care.

## Supplementary Information

Below is the link to the electronic supplementary material.


Supplementary Material 1



Supplementary Material 2



Supplementary Material 3



Supplementary Material 4



Supplementary Material 5



Supplementary Material 6



Supplementary Material 7



Supplementary Material 8



Supplementary Material 9



Supplementary Material 10



Supplementary Material 11


## Data Availability

Data availabilityThe data that support the findings of this study are derived from a major science and technology project funded by the Anyang City Major Science and Technology Special Project (Grant No. 2025A02SF008). As the current work represents interim results of this ongoing project, the full dataset is subject to confidentiality requirements stipulated by the funding agency prior to the official project completion and public release. Therefore, the data are not publicly available at this time. Anonymized summary data and the analytical methods necessary to interpret, verify, and extend the findings are fully presented in the manuscript and supplementary materials. Requests for access to specific data may be considered on a case-by-case basis after the project’s official conclusion, and should be directed to the corresponding author.

## Declarations

### Transparency statement

The lead author affirms that this manuscript is an honest, accurate, and transparent account of the study; that no important aspects have been omitted; and that any discrepancies from the study as planned have been explained.
