# Supplementary material for: Implementation of an ai-enabled multimodal emergency care system is associated with improved sudden cardiac death rescue outcomes in anyang
Source: Sci Rep. 2026 Jul 6;16:27092. doi: 10.1038/s41598-026-61073-w (PMC13524940; doi:10.1038/s41598-026-61073-w)
Supplement: Supplementary file 1 [file 41598_2026_61073_MOESM10_ESM.docx]

**Table S5. Diagnostics for interrupted time series models**

| **Outcome** | **Durbin-Watson statistic** | **p-value (autocorrelation)** | **Seasonality test (Wald)** | **p-value** |
| --- | --- | --- | --- | --- |
| Median response time | 1.94 | 0.58 | χ²(11)=14.2 | 0.22 |
| Pre-hospital STEMI ID | 1.87 | 0.41 | χ²(11)=12.5 | 0.33 |
| ROSC rate for OHCA | 2.01 | 0.82 | χ²(11)=10.9 | 0.45 |

*Interpretation: No significant residual autocorrelation or seasonality detected in any outcome. Models with monthly indicators did not significantly improve fit over linear time trend.*
