# Supplementary material for: Implementation of an ai-enabled multimodal emergency care system is associated with improved sudden cardiac death rescue outcomes in anyang
Source: Sci Rep. 2026 Jul 6;16:27092. doi: 10.1038/s41598-026-61073-w (PMC13524940; doi:10.1038/s41598-026-61073-w)
Supplement: Supplementary file 2 [file 41598_2026_61073_MOESM11_ESM.docx]

**Table S6. Real-time operational performance of AI alerts during post-implementation period (July–December 2023, n=621 calls)**

| **Metric** | **Deterioration warning model** | **STEMI detection model** |
| --- | --- | --- |
| Alert threshold | Risk score ≥65 | Probability >0.5 |
| Number of alerts (n, % of all calls) | 84 (13.5%) | 40 (6.4%)* |
| True positives (n) | 34 | 32 |
| False positives (n) | 50 | 8 |
| Sensitivity (%) | 74 (validation) / 71 (operational) | 92 (validation) / 94 (operational) |
| Specificity (%) | 91 (validation) / 89 (operational) | 96 (validation) / 95 (operational) |
| Positive predictive value (PPV, %) | 41% (34/84) | 80% (32/40)* |
| Negative predictive value (NPV, %) | 97% | 99% |
| False positive rate (per 100 calls) | 8.1% (50/621) | 1.3% (8/621) |

*Note: *STEMI alerts are triggered only for patients with ECG acquisition; denominator is 500 calls with ECG data. Operational sensitivity/specificity calculated against final hospital diagnosis.*
