# Supplementary material for: Implementation of an ai-enabled multimodal emergency care system is associated with improved sudden cardiac death rescue outcomes in anyang
Source: Sci Rep. 2026 Jul 6;16:27092. doi: 10.1038/s41598-026-61073-w (PMC13524940; doi:10.1038/s41598-026-61073-w)
Supplement: Supplementary file 3 [file 41598_2026_61073_MOESM1_ESM.docx]

**Supplementary Table S1. Baseline Characteristics of Study Population by Period**

| Characteristic | Pre-Implementation (n=587) | Post-Implementation (n=621) | p-value |
| --- | --- | --- | --- |
| Age, median (IQR), years | 67 (54-78) | 68 (55-79) | 0.42 |
| Male, n (%) | 340 (58) | 366 (59) | 0.76 |
| Urban cases, n (%) | 247 (42) | 273 (44) | 0.51 |
| Rural cases, n (%) | 340 (58) | 348 (56) | 0.51 |
| OHCA cases, n | 112 | 126 | 0.38 |
| Confirmed STEMI cases, n | 80 | 82 | 0.91 |
| Call-to-arrival time, median (IQR), minutes | 9.8 (7.2-13.1) | 6.7 (5.1-9.0) | <0.001 |

*Abbreviations: IQR, interquartile range; OHCA, out-of-hospital cardiac arrest; STEMI, ST-elevation myocardial infarction.*

**Supplementary Table S1. Baseline Characteristics of Study Population by Period.***Comparison of demographic and clinical characteristics of emergency cases between the pre-implementation (January–December 2022) and post-implementation (July–December 2023) periods. No significant differences were observed between the two periods in age, sex, urban/rural distribution, number of OHCA cases, or number of confirmed STEMI cases, indicating comparability of the study populations. The significant difference in response time is attributable to the system intervention rather than population characteristics. IQR: interquartile range; OHCA: out-of-hospital cardiac arrest; STEMI: ST-elevation myocardial infarction.
