# Supplementary material for: Implementation of an ai-enabled multimodal emergency care system is associated with improved sudden cardiac death rescue outcomes in anyang
Source: Sci Rep. 2026 Jul 6;16:27092. doi: 10.1038/s41598-026-61073-w (PMC13524940; doi:10.1038/s41598-026-61073-w)
Supplement: Supplementary file 4 [file 41598_2026_61073_MOESM2_ESM.docx]

**Supplementary Methods: Detailed Description of AI Model Development, Feature Selection, and Validation Procedures.**

**Data Source and Cohort Construction:** Retrospective data from 5,247 emergency cases treated at participating hospitals between 2019 and 2021 were used. Inclusion criteria: (1) age ≥18 years; (2) transported by EMS to the emergency department; (3) complete records of key variables (vital signs, ECG, outcomes). Exclusion criteria: (1) declared dead before hospital arrival; (2) traumatic cardiac arrest; (3) >20% missing data.

**Feature Engineering:** A total of 47 candidate features were included: (1) **Demographics** (age, sex); (2) **Vital signs** (systolic/diastolic blood pressure, heart rate, respiratory rate, oxygen saturation, temperature) and their temporal trends (rate of change over preceding 30 minutes); (3) **ECG features** (heart rate, QRS duration, QTc interval, ST elevation/depression, pathological Q waves, and 8 other automated measurements); (4) **Laboratory tests** (point-of-care troponin, CK-MB, electrolytes, glucose, lactate); (5) **Medical history** (hypertension, diabetes, coronary artery disease, heart failure, stroke, chronic kidney disease); (6) **Scene characteristics** (response time, location type (urban/rural), witness status, bystander CPR). Continuous variables were standardized; categorical variables were one-hot encoded.

**Model Development and Validation:** (1) **Deterioration Warning Model:** Gradient boosting machines (XGBoost) were used to predict cardiac arrest, shock, or unplanned ICU admission within 6 hours. Ten-fold cross-validation with grid search was used for hyperparameter tuning (tree depth, learning rate, subsampling ratio). Performance was evaluated using AUC, sensitivity, specificity, and calibration plots. (2) **STEMI Detection Model:** A convolutional neural network (CNN) was applied to 12-lead ECG signals. Input was raw ECG signals (10 seconds, 500Hz sampling); output was probability of STEMI. Ten-fold cross-validation was used, with performance evaluated by AUC, sensitivity, and specificity. Both models underwent calibration assessment (Hosmer-Lemeshow test) and decision curve analysis to evaluate clinical net benefit.
