# Supplementary material for: Implementation of an ai-enabled multimodal emergency care system is associated with improved sudden cardiac death rescue outcomes in anyang
Source: Sci Rep. 2026 Jul 6;16:27092. doi: 10.1038/s41598-026-61073-w (PMC13524940; doi:10.1038/s41598-026-61073-w)
Supplement: Supplementary file 5 [file 41598_2026_61073_MOESM3_ESM.docx]

**Supplementary Figure S2. Structure of the Distributed Multi-Source Data Management Module.** Illustrates the design of the distributed data management module, including the client, namespace manager, storage capacity manager, metadata service, data nodes (object block sets), and data consistency service. This architecture enables efficient storage, retrieval, and real-time synchronization of multi-source medical data, providing a reliable data foundation for AI algorithms.
