# Supplementary material for: Implementation of an ai-enabled multimodal emergency care system is associated with improved sudden cardiac death rescue outcomes in anyang
Source: Sci Rep. 2026 Jul 6;16:27092. doi: 10.1038/s41598-026-61073-w (PMC13524940; doi:10.1038/s41598-026-61073-w)
Supplement: Supplementary file 6 [file 41598_2026_61073_MOESM4_ESM.docx]

**Supplementary Figure S3. Functional Distribution of Diagnostic Equipment Across the Emergency Care Continuum.** Illustrates the deployment of diagnostic equipment—including POCT devices, handheld ultrasound, cloud-based ECG systems, AEDs, and video laryngoscopes—at three key nodes along the emergency care continuum: (1) **Emergency Scene:** Portable devices enable rapid assessment by first responders; (2) **Ambulance:** Vehicle-mounted professional equipment (on-board CT, ECMO, ventilator, automated CPR machine) enables en-route examination and treatment, while serving as a data relay station transmitting real-time information to the receiving hospital; (3) **In-Hospital:** Pre-hospital data are received and integrated, ensuring continuity and completeness of medical services. 5G technology ensures data transmission, while smart monitoring ensures device availability and information security.
