# Supplementary material for: Implementation of an ai-enabled multimodal emergency care system is associated with improved sudden cardiac death rescue outcomes in anyang
Source: Sci Rep. 2026 Jul 6;16:27092. doi: 10.1038/s41598-026-61073-w (PMC13524940; doi:10.1038/s41598-026-61073-w)
Supplement: Supplementary file 7 [file 41598_2026_61073_MOESM5_ESM.docx]

**Supplementary Figure S1. Detailed Technical Architecture Diagram.** Illustrates the complete system technology stack, including the data middle platform, infrastructure components (nacos, redis, rabbitmq, elasticsearch, etc.), front-end and back-end application integration, and interfaces with hospital information systems (HIS, LIS, PACS, EMR) and third-party platforms (national platforms, specialized disease data centers). This diagram presents the underlying technical architecture supporting the AI decision platform.
