# Supplementary material for: Implementation of an ai-enabled multimodal emergency care system is associated with improved sudden cardiac death rescue outcomes in anyang
Source: Sci Rep. 2026 Jul 6;16:27092. doi: 10.1038/s41598-026-61073-w (PMC13524940; doi:10.1038/s41598-026-61073-w)
Supplement: Supplementary file 8 [file 41598_2026_61073_MOESM6_ESM.docx]

**STROBE Checklist (Supplementary Table S3)**

**STROBE Statement—Checklist of items that should be included in reports of cohort studies**

| **Section/Topic** | **Item No.** | **Recommendation** | **Where reported (page/line)** |
| --- | --- | --- | --- |
| **Title and abstract** | 1 | (a) Indicate the study’s design with a commonly used term in the title or abstract | Title: “quasi-experimental before-and-after study”; Abstract: “before-and-after study” |
|  |  | (b) Provide in the abstract an informative and balanced summary of what was done and what was found | Abstract: full summary provided |
| **Introduction** |  |  |  |
| Background/rationale | 2 | Explain the scientific background and rationale for the investigation being reported | Introduction, pages 3-4 |
| Objectives | 3 | State specific objectives, including any prespecified hypotheses | Introduction, last paragraph; Methods, first paragraph |
| **Methods** |  |  |  |
| Study design | 4 | Present key elements of study design early in the paper | Methods 2.1, page 5 |
| Setting | 5 | Describe the setting, locations, and relevant dates, including periods of recruitment, exposure, follow-up, and data collection | Methods 2.1, page 5 |
| Participants | 6 | (a) Give the eligibility criteria, and the sources and methods of selection of participants | Methods 2.1, page 5; 2.3, page 7 |
|  |  | (b) For cohort studies, method of follow-up | Not applicable (retrospective data from EMS logs) |
| Variables | 7 | Clearly define all outcomes, exposures, predictors, potential confounders, and effect modifiers. Give diagnostic criteria, if applicable | Methods 2.3 (AI outcomes definition), 2.5 (primary outcomes) |
| Data sources/measurement | 8 | For each variable of interest, give sources of data and details of methods of assessment (measurement). Describe comparability of assessment methods if there is more than one group | Methods 2.5, page 8-9 |
| Bias | 9 | Describe any efforts to address potential sources of bias | Methods 2.5: blinded data abstractors, interobserver agreement (kappa=0.92); ITS analysis; page 8-9 |
| Study size | 10 | Explain how the study size was arrived at | All eligible cases during study periods; n provided |
| Quantitative variables | 11 | Explain how quantitative variables were handled in the analyses. If applicable, describe which groupings were chosen and why | Methods 2.5: medians with IQR; ITS with monthly aggregation |
| Statistical methods | 12 | (a) Describe all statistical methods, including those used to control for confounding | Methods 2.5: Mann-Whitney U, chi-square, ITS, adjustment for temperature/staffing |
|  |  | (b) Describe any methods used to examine subgroups and interactions | Subgroup analyses by urban/rural, witnessed arrest, shockable rhythm |
|  |  | (c) Explain how missing data were addressed | Supplementary Methods: multiple imputation for missing values |
|  |  | (d) If applicable, describe analytical methods taking account of sampling strategy | Not applicable |
|  |  | (e) Describe any sensitivity analyses | ITS analysis, adjustment for confounders |
| **Results** |  |  |  |
| Participants | 13 | (a) Report numbers of individuals at each stage of study | Results: 587 pre, 621 post; total 1,208 |
|  |  | (b) Give reasons for non-participation at each stage | All consecutive cases; no refusal |
|  |  | (c) Consider use of a flow diagram | Not included but can be added |
| Descriptive data | 14 | (a) Give characteristics of study participants (e.g., demographic, clinical, social) and information on exposures and potential confounders | Supplementary Table S1 |
|  |  | (b) Indicate number of participants with missing data for each variable of interest | <20% missing; addressed by imputation |
|  |  | (c) Summarise follow-up time (e.g., average and total amount) | 6 months post-implementation |
| Outcome data | 15 | Report numbers of outcome events or summary measures | Table 1; Results 3.1 |
| Main results | 16 | (a) Give unadjusted estimates and, if applicable, confounder-adjusted estimates and their precision (e.g., 95% CI). Make clear which confounders were adjusted for and why they were included | Table 1 unadjusted; ITS provides adjusted level change with 95% CI |
|  |  | (b) Report category boundaries when continuous variables were categorized | Not applicable (continuous as continuous) |
|  |  | (c) If relevant, consider translating estimates of relative risk into absolute risk for a meaningful time period | Not applicable |
| Other analyses | 17 | Report other analyses done—e.g., analyses of subgroups and interactions, and sensitivity analyses | Subgroup analyses (rural/urban, witnessed arrest); ITS sensitivity analyses |
| **Discussion** |  |  |  |
| Key results | 18 | Summarise key results with reference to study objectives | Discussion, first paragraph |
| Limitations | 19 | Discuss limitations of the study, taking into account sources of potential bias or imprecision. Discuss both direction and magnitude of any potential bias | Discussion 4.4, pages 12-13 |
| Interpretation | 20 | Give a cautious overall interpretation of results considering objectives, limitations, multiplicity of analyses, results from similar studies, and other relevant evidence | Discussion 4.1-4.6 |
| Generalisability | 21 | Discuss the generalisability (external validity) of the study results | Discussion 4.4 (LMICs), 4.5 |
| **Other information** |  |  |  |
| Funding | 22 | Give the source of funding and the role of the funders for the present study and, if applicable, for the original study on which the present article is based | Funding section |

*Note: An Explanation and Elaboration article discusses each checklist item and gives methodological background and published examples of transparent reporting. The STROBE checklist is best used in conjunction with this article (freely available on the Web sites of PLoS Medicine, Annals of Internal Medicine, and Epidemiology).*
