# Supplementary material for: Implementation of an ai-enabled multimodal emergency care system is associated with improved sudden cardiac death rescue outcomes in anyang
Source: Sci Rep. 2026 Jul 6;16:27092. doi: 10.1038/s41598-026-61073-w (PMC13524940; doi:10.1038/s41598-026-61073-w)
Supplement: Supplementary file 9 [file 41598_2026_61073_MOESM7_ESM.docx]

**Supplementary Table S2. AI Algorithm Performance Metrics During Validation and Operation**

| Algorithm | Validation AUC (95% CI) | Operational Sensitivity | Operational Specificity | PPV |
| --- | --- | --- | --- | --- |
| Deterioration warning | 0.89 (0.86-0.92) | 84% | 91% | 41% |
| STEMI detection | 0.92 (0.89-0.94) | 92% | 96% | 78% |

*Abbreviations: AUC, area under the receiver operating characteristic curve; CI, confidence interval; PPV, positive predictive value; STEMI, ST-elevation myocardial infarction.*

**Supplementary Table S2. AI Algorithm Performance Metrics During Validation and Operation.** Performance of the two core AI algorithms—deterioration warning model and STEMI detection model—during 10-fold cross-validation (validation set) and actual operation (July–December 2023). The deterioration warning model achieved a validation AUC of 0.89 (95% CI: 0.86–0.92), with operational sensitivity of 84%, specificity of 91%, and positive predictive value of 41%. The STEMI detection model achieved a validation AUC of 0.92 (95% CI: 0.89–0.94), with operational sensitivity of 92%, specificity of 96%, and positive predictive value of 78%. Both algorithms demonstrated consistent performance between validation and operation, indicating good generalizability and operational stability. AUC: area under the curve; CI: confidence interval; PPV: positive predictive value.
