# Supplementary material for: Implementation of an ai-enabled multimodal emergency care system is associated with improved sudden cardiac death rescue outcomes in anyang
Source: Sci Rep. 2026 Jul 6;16:27092. doi: 10.1038/s41598-026-61073-w (PMC13524940; doi:10.1038/s41598-026-61073-w)
Supplement: Supplementary file 10 [file 41598_2026_61073_MOESM8_ESM.docx]

**Table S4. Sensitivity analyses for interrupted time series models of primary outcomes**

| **Outcome** | **Sensitivity specification** | **Coefficient (level change)** | **95% CI** | **p-value** |
| --- | --- | --- | --- | --- |
| Median response time (minutes) | Primary mode | -2.8 | -3.7 to -1.9 | <0.001 |
|  | 3-month lagged intervention | -2.6 | -3.5 to -1.7 | <0.001 |
|  | Excluding first post-month | -2.9 | -3.8 to -2.0 | <0.001 |
|  | Adjusted for EMS call volume | -2.7 | -3.6 to -1.8 | <0.001 |
| Pre-hospital STEMI ID (%) | Primary model | +22 | +13 to +31 | <0.01 |
|  | 3-month lagged intervention | +20 | +11 to +29 | <0.01 |
|  | Excluding first post-month | +23 | +14 to +32 | <0.01 |
|  | Adjusted for EMS call volume | +21 | +12 to +30 | <0.01 |
| ROSC rate for OHCA (%) | Primary model | +12 | +5 to +19 | <0.01 |
|  | 3-month lagged intervention | +11 | +4 to +18 | <0.02 |
|  | Excluding first post-month | +13 | +6 to +20 | <0.01 |
|  | Adjusted for EMS call volume | +12 | +5 to +19 | <0.01 |

*Notes: Primary model includes terms for time, intervention, and time after intervention. Lagged model assumes full effect only after 3 months. Excluding first post-month removes July 2023 data to account for wash-in. Adjusted model includes monthly EMS call volume as a covariate.*
