# Supplementary material for: Implementation of an ai-enabled multimodal emergency care system is associated with improved sudden cardiac death rescue outcomes in anyang
Source: Sci Rep. 2026 Jul 6;16:27092. doi: 10.1038/s41598-026-61073-w (PMC13524940; doi:10.1038/s41598-026-61073-w)
Supplement: Supplementary file 11 [file 41598_2026_61073_MOESM9_ESM.docx]

**Supplementary Table S7. System uptake and response time reduction by county**

| County | Urban/rural classification | POCT usage (% of runs) | AI alert acknowledgment rate (%) | Median response time reduction (minutes) |
| --- | --- | --- | --- | --- |
| Hua County | Urban core | 96% | 91% | 2.8 |
| Neihuang County | Mixed | 92% | 87% | 3.5 |
| Tangyin County | Rural | 89% | 84% | 4.2 |

*Note: This table presents county-level system usage metrics and the associated improvement in median emergency response time following implementation of the AI-enabled platform. Hua County represents the urban core, Neihuang County a mixed urban-rural area, and Tangyin County the predominantly rural catchment. Although POCT usage and AI alert acknowledgment rates were slightly lower in the rural county (Tangyin), the reduction in response time was largest there (4.2 minutes vs. 2.8 minutes in urban Hua County), suggesting that the system may benefit rural areas most despite marginally lower fidelity.*
